# Vulnerability of the peatland carbon sink to sea-level rise

**DOI:** 10.1038/srep28758

**Published:** 2016-06-29

**Authors:** Alex Whittle, Angela V. Gallego-Sala

**Affiliations:** 1 Department of Geography, University of Exeter, Exeter, EX4 4RJ, UK

## Abstract

Freshwater peatlands are carbon accumulating ecosystems where primary production exceeds organic matter decomposition rates in the soil, and therefore perform an important sink function in global carbon cycling. Typical peatland plant and microbial communities are adapted to the waterlogged, often acidic and low nutrient conditions that characterise them. Peatlands in coastal locations receive inputs of oceanic base cations that shift conditions from the environmental optimum of these communities altering the carbon balance. Blanket bogs are one such type of peatlands occurring in hyperoceanic regions. Using a blanket bog to coastal marsh transect in Northwest Scotland we assess the impacts of salt intrusion on carbon accumulation rates. A threshold concentration of salt input, caused by inundation, exists corresponding to rapid acidophilic to halophilic plant community change and a carbon accumulation decline. For the first time, we map areas of blanket bog vulnerable to sea-level rise, estimating that this equates to ~7.4% of the total extent and a 0.22 Tg yr^−1^ carbon sink. Globally, tropical peatlands face the proportionally greatest risk with ~61,000 km^2^ (~16.6% of total) lying ≤5 m elevation. In total an estimated 20.2 ± 2.5 GtC is stored in peatlands ≤5 m above sea level, which are potentially vulnerable to inundation.

Peatland ecosystems play an important role in the global carbon cycle^1^,^2^,^3^, containing around 600 Gt of carbon sequestered persistently since the Last Glacial Maximum^4^. They are also biodiverse and help support the livelihoods of communities worldwide^5^. Accumulation of carbon-rich peat deposits occurs when net primary production (NPP) exceeds the rate of decomposition in the peat matrix, which is supressed by anoxic and often acidic conditions^6^. Whilst definitions vary contextually^7^, peatlands can be geographically categorised as either tropical (occurring between 30°S–30°N) or high-latitude (boreal and temperate regions). High latitude peatlands are more extensive; they represent 90% of the total peatland area and contain 562 Gt of carbon compared to 50 Gt in tropical peatlands^2^.

The continuing existence of peatland ecosystems is endangered by climate change and direct anthropogenic pressures (e.g. drainage, pollution and extraction). Rising global sea levels are also a possible threat^8^,^9^ and may drive future releases of stored carbon^10^,^11^. Between 1901–2010 global average sea level rose by 0.19 ± 0.02 m^12^. Projections of 21^st^ century global mean sea level show continuing rise at rates exceeding the observed 1971–2010 average (2.0 ± 0.3 mm yr^−1^) at all representative concentration pathway scenarios^13^. As sea levels rise, the frequency and magnitude of salt-transfer to peatlands will increase, threatening those at very low elevation. Palaeoenvironmental evidence indicates that freshwater peatlands have undergone similar changes in the past with records of both marine transgressions under rising Holocene sea levels^14^, as well as coastward colonisation in areas of relative sea-level fall^15^.

Blanket bogs are distinctive ecosystems occupying the coastal niches (hyperoceanic regions) of the high-latitude peatland space, which makes them especially vulnerable to sea-level change. Their occurrence in such regions is driven by unique climatic requirements of high year-round rainfall and low summer temperatures^16^. *Sphagnum* mosses have low salt tolerance and typically dominate understorey vegetation assemblages. They are important in the accumulation of carbon and thought to hold more organic C than any other plant genus^6^. As for all peatlands, viable long-term persistence of blanket bog is dependent on the maintenance of a positive carbon balance, i.e. draw-down of atmospheric carbon must exceed the quantity lost through numerous export pathways^17^. Despite the occurrence of blanket bogs within coastal interface zones, the potential for enhanced oceanic salt inputs to perturb these ecosystems and their carbon balance remains understudied. Indeed no estimates for the areal extent of threatened blanket bog currently exist.

Previous observational studies on the ecological impact of oceanic salinity on peatlands have focused on the lithotrophic-thalassotrophic gradient, encompassing responses to variable deposition of oceanic ions (S^2−^, Cl^−^, Mg^2+^, etc.) at the landscape scale^7^,^18^,^19^. Specifically we investigate, at the local-scale, the impacts of direct salt-water delivery from the ocean to blanket bogs via inundation. We interpret a modern blanket bog salinity gradient as a spatial analogue for the ecosystem response to increased salt inputs in order to forecast the potential effects of future inundation on the ecosystem and carbon balance. We combine this empirical approach with mapping of peatland areas at risk globally in order to consider the future longevity of coastal blanket bogs and their associated carbon sink function.

## Study Area

**Figure 1:**
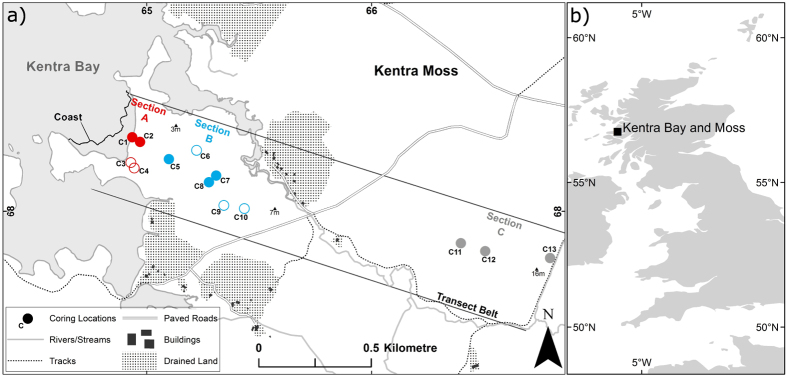
(**a**) Arrangement of the transect belt, and location of cores, divided into three sections based on vegetation. Section A (C1–C4: Red) inundated regularly, Section B (C5–C10: Blue), and Section C (C11–C13: Grey). Filled markers indicate cores used to calculate carbon accumulation and assess peat properties. Hollow markers indicate cores used to assess peat properties only. Spot height markers indicate elevation above sea level. (**b**) Location of the site at Kentra Bay and Moss, Northwest Scotland within the United Kingdom. Produced using ESRI ArcMap 10.1. (http://www.esri.com/). Contains OS data © Crown copyright (2015).

The blanket bog to coastal marsh transition at Kentra Bay, Northwest Scotland (56°45′N, 5°50′W) (Fig. 1; Supplementary material 1) provides several advantages for studying the impact of salinity on the ecosystem and carbon accumulation. The meso-tidal regime of the bay and its high salinity (33%^20^) waters produce a measurable salinity gradient across the coastal interface. The bay is also sheltered from high-energy waves protecting the blanket bog from erosion and allowing it to exist close to sea level. Additionally, the site permits establishment of a continuous transect focused on a small region with minimal macro-topographic variability thereby controlling against climatic effects on peat development.

Peat initiation at the site occurred approximately 8,000 years before present (8 kyr B.P.), within topographic surface depressions of a fluvioglacial outwash fan deposited during the Loch Lomond stadial (11–10 kyr B.P.)^21^,^22^. Blanket bog, which now covers the site, expanded continually toward the coast to its present limit as sea levels fell throughout the Holocene^23^. The bog surface has a well-developed patterning of elongate surface pools and ridges aligned with contours of the underlying outwash fan which drop gently towards the coast^21^,^22^, where a narrow strip of salt marsh has established.

Vegetation is characteristic of western blanket bog^22^, with abundant *Sphagnum* spp. and distinctive *Racomitrium lanuginosum* hummocks. Summer water surplus is 500 mm^22^ with an average yearly total of ~1700 mm distributed evenly^24^. Rainfall of more than 1 mm occurs on average 192 days per year; mean annual temperature is 7.9 °C, and mean temperature of the warmest month is 13.6 °C^24^. The site has not been substantially altered by anthropogenic activity, although some peat extraction has occurred in peripheral areas and a road runs across the site. The transect we studied represents a rare uninterrupted succession from salt marsh to ombrotrophic bog habitat, and therefore provides a unique opportunity to directly assess the impacts of salt-flux.

## Results

### Surface Water Salinity Gradient

**Figure 2:**
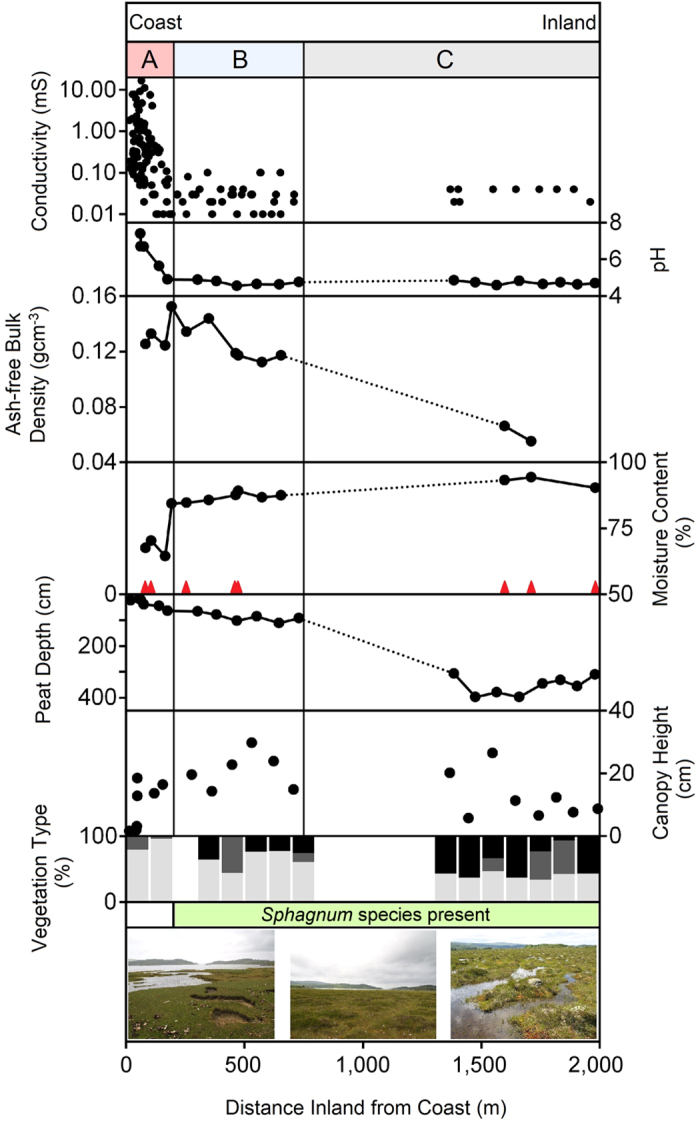
Variables measured at the land-sea transect at Kentra Moss. Sections A–C correspond to Fig.1 and are divided by dominant vegetation to aid interpretation. Dominant species are; *Armeria maritima* and *Plantago* spp. (Section A), *Ericaceae, Eriophorum* spp. and *Molinia caerulea* (Section B) and, *Racomitrium lanuginosum* and *Sphagnum* spp. (Section C). Vegetation type, refers to percentage surface cover of *Sphagnum* spp. (black), other bryophytes (dark-grey) and vascular plants (light-grey). Red triangles indicate the positions within the transect of cores analysed for carbon accumulation rate (Fig. 3).

Conductivity and pH measurements of surface waters identify a gradient of oceanic base cations along the transect belt at Kentra Bay (Supplementary material 2), which we separate into three sections (A–C) according to dominant vegetation to assess the effect of salinity on: peat properties (Fig. 2) and carbon accumulation rates. High pH and conductivity values between 0–200 m from the coast are due to salt input from either tidal inundation or less frequent storm surges. During high tides salt water infiltrates inland via a dendritic system of channels which drain from the bog at low tide (Section A, salt marsh). Beyond 200 m inland, above the maximum inundation level, a rapid transition to predominantly ombrotrophic conditions occurs (i.e. low conductivity and low pH). Here, limited salt deposition from; sea-spray during storms, fog and precipitation, nourishes the ecosystem due to its close coastal proximity (Section B, coastal bog). At the distal end of the transect (2 km inland, Section C, blanket bog) true ombrotrophic conditions prevail, due to isolation from the coast, although precipitation and fog deposition of salt enhance inputs relative to blanket bog in more isolated upland areas.

### Peat Properties

The thirteen monolith-cores analysed (C1–C13, Fig. 1) provide an insight into patterns of variability in peat composition across the blanket bog to coast interface. Peat formed at the coast (Section A), displays the highest average bulk density and lowest carbon content relative to peat formed where inundation does not occur (Table 1). Down-core variability in bulk density is more pronounced in the inundated peat, and cores taken inland where peat was composed mostly of decaying *Sphagnum* tissue show only minor variability of bulk density with depth. Maximum average carbon content is 51.47% (C9, Section B) and the lowest is 19.19% (C1, Section A).

We use ash-free bulk density (Fig. 2) as a proxy for level of decomposition^25^,^26^. Results indicate that organic matter comprising peat in areas closer to the coast (Section A and B) is generally more decomposed than those isolated from the coast (Section C).

Average moisture content within each core mirrors the gradient of salinity across the site – characterised by an abrupt change from low to high moisture conditions at 200 m from the coast, where water chemistry measurements indicate the transition to ombrotrophic conditions.

### Vegetation

Considerable variation of the blanket bog surface was observed along the gradient of salinity (Fig. 2; Supplementary material 3). Frequently inundated areas (Section A) are characterised by a low diversity of small stature halophilic plant species (e.g. *Armeria maritima, Glaux maritima, Plantago maritima* and *Salicornia maritima*). Above the inundation level (Section B), vegetation changed rapidly from halophilic to acidophilic species. Here plant stature and diversity increases, with a dense canopy including *Agrostis capillaris*, *Molinia caerulea* and woody species (e.g. *Calluna vulgaris* and *Erica tetralix*). *Sphagnum* mosses and other bryophytes (notably *Racomitrium lanuginosum* as the main hummock species) dominate within Section C, accompanied by species adapted to blanket bog conditions including *Narthecium ossifragum, Drosera intermedia* as well as *Myrica gale*. At the distal end of the transect (2,000 m inland), where a complex system of pools have developed, species such as *Menyanthes trifoliata* are present.

We interpret canopy height as an indicator of NPP, with greater canopy height indicating higher productivity^27^. Canopy height is very low at the coast, peaks at ~500 m inland, before declining further inland (Fig. 2). Average canopy height also indicates the type of vegetation colonising the bog surface, since bryophytes are slow growing and of small stature compared with vascular plants. The dominance of slow growing bryophytes (including *Sphagnum* spp.) within Section C results in lesser input of organic carbon to the soil, whereas enhanced inputs occur where faster growing vascular species predominate.

The zonation of *Sphagnum* spp. along the transect coincides with surface water chemistry variation, with low pH values and conductivity corresponding to areas of *Sphagnum* spp. presence. Areas where *Sphagnum* spp. are present are also characterised by high soil moisture contents. *Sphagnum* spp. are absent between 0–200 m from the coast, with the exception of isolated areas where topography prevents inundation.

### Carbon Accumulation

**Figure 3:**
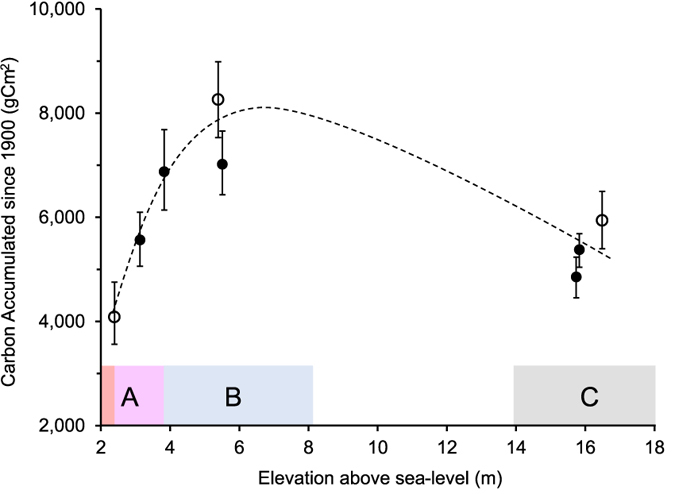
Carbon accumulation against elevation above sea level. Sections of variable salt input (A–C) correspond with Figs 1 and 2. Dark red indicates the maximum level of tidal inundation. Filled markers represent carbon accumulation calculated using ^210^Pb dating whilst hollow markers indicate results derived from Spheroidal Carbonaceous Particle dating. Error bars show uncertainty associated with sampling and dating accuracy.

Integration of carbon density and age-depth models from spheroidal carbonaceous particles (SCP) and ^210^Pb dating provides the rate and total carbon accumulation since 1900. Carbon accumulation results plotted against elevation allow direct assessment of the impact of inundation (Fig. 3). Greatest accumulation since 1900 (8,260 ± 730 gCm^−2^) was recorded within Section B at 5.4 m above sea level, where low-concentration salt input and no inundation occurs. Carbon accumulation where salt water inundation occurs regularly is substantially lower (4,135 ± 620 gCm^−2^). From 2–6 m above sea level (~0–500 m inland) total carbon accumulated since 1900 increases abruptly. Greatest peat depth occurs in Section C (Fig. 2) and generally declines toward the coast.

The individual datasets were brought together using a stepwise multivariate regression to understand whether total carbon accumulation since 1900 can be predicted from any of the measured variables. Conductivity first and then elevation were found to be the two statistically significant predictor variables. Conductivity presents a negative linear relationship to carbon accumulation since 1900 (p = 0.0057), while elevation presents a quadratic relationship with accumulation (Fig. 3). Overall the model fit was high (Adjusted R^2^ = 0.76) and also significant (F(2, 5) = 12.13, p = 0.012), suggesting that increases of salinity are likely to result in lower accumulation. Elevation is less significant (p = 0.013), but also exerts some control over the total carbon accumulated since 1900. The maximum accumulation values occur at mid elevation and we discuss the possible implications of this in the discussion section.

### Mapping low elevation peatlands

**Figure 4:**
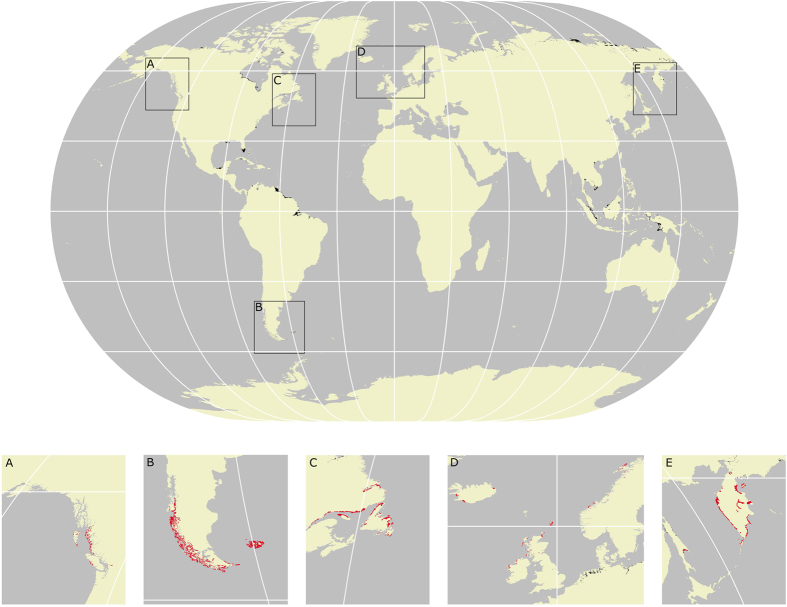
Map of global peatlands (black shading) lying at or below 5 m elevation. Insets (A–E) show the locations of low lying blanket bog areas (red shading). Produced using ESRI ArcMap 10.2. (http://www.esri.com/).

Global assessment of peatland areas vulnerable to sea-level rise revealed a disjunctive pattern (Fig. 4). Our analysis indicates that ca. 145,000 km^2^ of peatlands are located at or below 5 m elevation, in close agreement with an earlier estimate^10^.

Because blanket bogs are constrained climatically to hyperoceanic regions at high-latitude^16^, our mapping indicates that vulnerable areas occur within all of the major regions in which they are present. In South America, potentially threatened areas exist in western Patagonia and the Falkland Islands; in North America, on the coast of British Columbia and substantial areas in Newfoundland; and in Europe within Scotland, Iceland and Ireland. Vulnerable areas also exist around the coast of Kamchatka in Russia. According to our mapping, blanket bogs cover a total land area of 155,000 km^2^ of which ~7.4% is below 5 m elevation, equating to approximately 11,500 km^2^ of blanket bogs at risk.

The spatial investigation of regions at risk identified that approximately 16.6% of tropical peatlands (30°N–30°S of the equator) are situated at or below 5 m elevation and can therefore be considered vulnerable to future sea-level changes (Table 2). This equates to ca. 61,000 km^2^ and a store of 8.28 ± 1.0 Gt of carbon^2^. High latitude peatlands are proportionately less vulnerable by percentage with approximately 2% at or below 5 m. However, because the majority of peatlands occur at high latitude this equates to ~83,000 km^2^ and 12 ± 1.7 Gt of carbon^2^.

## Discussion

Hyperoceanic conditions and the influence of the Gulf Stream provide suitable bioclimatic space for blanket bog development at Kentra Moss, and therefore landscape-scale proximity to the Atlantic Ocean is an important influence on ecosystem functioning and carbon sequestration at the site. The focus of our work has been to investigate the impact of high concentration oceanic salt input on coastal blanket bogs at the sub-kilometre scale.

We attribute the conductivity gradient to the morphology of the site, because it allows oceanic salt deposition from both direct (inundation) and atmospheric (salt-spray, fog and precipitation) sources. When combined, these delivery mechanisms produce the salinity gradient, which we classified into three sections; (A) land inundated directly, (B) areas not inundated but receiving enhanced atmospheric salt input owing to coastal proximity, and (C) inland areas receiving lower concentrations due to increased isolation from the coast. Indeed, surface water pH across Kentra Moss (mean = pH 4.82) is higher than expected (<4) for ombrotrophic blanket bog^9^,^28^, indicative of continual oceanic base cation input. Similarly elevated pH values have been reported for coastal blanket bogs nearby in the North of Scotland^29^ and West Coast of Ireland^14^.

Surface water conductivity (a proxy for salinity) was identified as the most powerful explanatory variable in predicting carbon accumulation at Kentra Moss, indicating that oceanic salt input exerts control on blanket bog ecosystem functioning. Since carbon accumulation rates reflect the balance of carbon inputs and outputs, the effects of salt on both the ecosystem NPP and decomposition processes are needed to explain our results.

*Sphagnum* mosses actively create conditions of acidity, low-nutrients and soil anoxia, allowing them to outcompete vascular plants^30^. Such conditions were found in areas of lower salt input where *Sphagnum* spp. dominate (Section C). Importantly for carbon accumulation, the release of phenolic compounds from *Sphagnum* inhibit microbial decomposition^31^ and recalcitrant tissue slows the decay of its biomass relative to that of vascular plants^30^, facilitating the net accumulation of carbon. Whilst *Sphagnum* biomass is generally not the largest component of input NPP^32^, a greater percentage of its biomass resists decay in the surface aerobic acrotelm and is transmitted to long-term storage in the anaerobic catotelm. The expansion of *Sphagnum* spp. coastward at Kentra is constrained by high-concentration salt input from inundation, supporting recent work suggesting their low tolerance to salinity^33^.

Mineral enrichment from atmospheric salt delivery in intermediate areas (Section B) moderate the low nutrient and acidic conditions, favouring the more productive (and faster decomposing) vascular plants which outcompete *Sphagnum* spp. to colonise the surface. This effect on ecosystem functioning can be considered analogous to the impacts of Nitrogen deposition from industrial and agricultural pollution. Similarly, Nitrogen deposition also leads to vascular plant dominance^34^ and to a shift in the decomposition pathway from methane to nitrate or sulphate as the final electron acceptor^35^. We therefore expect higher decomposition rates in areas of strong salt influence, and this is corroborated by our ash-free bulk density measurements, which are lowest inland.

Decomposition processes are also strongly affected by the position of the water table. Lower soil moisture content at the coast represents the hydrological connectivity between tidal and water-table fluctuation within this section of the transect. At low tide the water-table drops switching decomposition of organic matter from the slower anaerobic to the faster aerobic pathway, increasing microbial respiration of input biomass and reducing carbon accumulation. Furthermore, there may be an unquantified amount of carbon loss via particulate organic carbon (POC) and dissolved organic carbon (DOC) transportation during the downward tidal water movement. Through ombrotrophication (blanket bog expansion), sections B and C are isolated from the water table and hence anaerobic conditions persist close to the surface.

We attribute peak carbon accumulation at intermediate elevation and distance from the coast partially to the enhanced rates of biomass input associated with dominant vascular plant colonisation and partially to the age of the deposits and the ecosystem maturity effect^36^. Although high decomposition has been measured in intermediate areas, the high biomass input increases the carbon accumulation rate. Where *Sphagnum* dominates, at the inland end of the transect, decomposition is minimal but biomass production is slow resulting in lower carbon accumulation. Carbon accumulation was lowest at the coastal interface because productivity was minimal and decomposition was high.

Over long time-scales, succession from saltmarsh to blanket bog requires the vertical growth of peat deposits and subsequent disconnection from the water table (ombrotrophication). Section C at Kentra Moss represents mature blanket bog and Section B represents a prior successional stage modulated by salt input, because coastward expansion has exploited areas exposed by falling Holocene sea levels. Palaeoenvironmental work at the site identified salt-marsh sediments underlying the blanket bog^21^, indicating that conditions equivalent to section A are the primary successional stage in the development of coastal blanket bog of this type. Indeed our results suggest that an environmental tipping point exists, governed by salt-water inundation, which limits blanket bog expansion coastward by facilitating high decomposition and inhibiting typical plant communities. Under the Holocene sea-level decline at Kentra Moss, blanket bog has expanded coastward, as new areas beyond the inundation level have been exposed, allowing succession of vegetation and increasing suppression of decomposition processes. This interpretation is corroborated by our peat depth measurements, which imply that peat initiation occurred earlier inland and spread toward the present coastline in agreement with previous studies^21^,^22^.

Modelling of near-future sea-level rise (Supplementary material 4) indicates that Kentra Moss is not immediately vulnerable, however our results from the salinity transect indicate that other similar coastal bogs that do experience sea intrusion may be under threat in the future. We infer a model of peatland ecosystem decline where increasing sea-level tips the salt concentrations over the tolerance threshold of freshwater peat-forming vegetation (Section B) allowing salt-marsh vegetation (Section A) to colonise the peat substrate left behind. This process is manifest on the West coast of Ireland where salt marshes currently colonise a peat substrate accumulated under freshwater conditions before a rise in sea-level^37^. If sea-level rise continued such conditions would precede states of total inundation, corresponding to complete loss of vegetation. Under such scenarios the areal extent of currently healthy blanket bog will decline along with their global contribution to the budget of atmospheric carbon sinks.

The decline in carbon sink functioning attributed to salt-water inundation at Kentra Moss highlighted the need for an estimation of the area of low-elevation coastal blanket bogs potentially vulnerable in the future. We then expanded our analysis to incorporate peatlands of all types. Our estimates also address an improvement of using peatland specific mapping suggested in previous work^10^. To make our estimates comparable we applied the same (Hydro1k) digital elevation model (DEM) to derive land-surface heights and made estimates of peatland loss under the same +5 m sea-level scenario.

Many of the blanket bog areas identified as at risk from rising sea level are also vulnerable to climatic changes in the future^16^. In total 11,500 km^2^ (~7.4% of the global total) of blanket bogs are located either at or below 5 m elevation. This equates to ca. 2.1 ± 0.34 Gt of stored carbon and a yearly carbon sink of 0.22 Tg.

Although our analysis was primarily aimed at quantifying blanket bog areas at risk, our global mapping shows that high-latitude peatlands, such as the blanket bog at Kentra Moss, are proportionately less vulnerable to sea-level change. This is attributed to their colonisation of more recently deglaciated land, areas now mostly experiencing isostatic rebound which contributes to low or negative rates of relative sea-level rise. In such areas active carbon accumulation and expansion coastward is likely, offsetting losses to rising sea levels elsewhere. The threat of inundation facing peatlands in the tropics is proportionately greater.

We identified Southeast Asia as the geographical region where the largest areas of peatland are threatened by sea-level change in the future (Supplementary material 5), supporting previous analysis^10^. Low-lying tropical peatlands are usually wooded (including mangroves) so the impacts of salinity in these areas may be very different from those at Kentra Bay. However, it is likely that in this region the threat of salt-water inundation will be exacerbated by recent land use changes^38^ (e.g. forest clearance and drainage for oil palm plantations) leading to rapid oxidation and wild-fires that eventually result in land subsidence^39^. Tropical peats subside rapidly after drainage by ~1.5 m after 5 years with continued loss at rates of ~3–5 cm yr^−1^, equivalent to surface lowering of 4–5 m over 100 years^40^. Similarly to the saltmarsh sediments underlying the blanket bog at Kentra Moss, tropical peat domes often originate from mangroves and floodplains either at or below sea level. Due to the depths of these peat deposits (mean >5 m and up to 20 m or more^40^,^41^) the subsidence of coastal lowland peatlands is a significant mechanism enhancing the threat of inundation that they face.

The risk of tropical peat collapse following saltwater intrusion has been suggested by research conducted in the Everglades National Park (USA), which is also highlighted as a high-risk region by our mapping (Fig. 4). A positive feedback may result from the intrusion of saltwater, a symptom of rising sea levels or increased storm surge frequency, causing sawgrass dieback and subsequent degradation of freshwater peat. Surface lowering allows water depths to rise inhibiting the establishment of plants, and high salinity means that soil is only stabilised after salt tolerant mangrove colonisation^42^.

Importantly ocean inundation of the peatlands does not necessarily imply that the carbon they store would be immediately oxidised and released as CO_2_ to the atmosphere. Oxidation of carbon after inundation is one possibility, but if peat experienced relatively rapid burial under sediment, then carbon stores could be maintained^43^, converted into lignite and eventually coal. In South-west England, peat substrates buried beneath beach deposits have recently been exposed, indicating that complete loss of carbon is not an inevitable consequence of inundation. Such features are widespread elsewhere in the United Kingdom^44^. However, whilst a peat substrate may remain intact post-inundation, loss of the peatland as an ecosystem is inevitable. Possible carbon losses, and the changing accumulation rates of inundated coastal peatlands, therefore represent a component of direct carbon flux laterally across the land-ocean aquatic continuum, which is currently uncertain.

Equally, predictions of short-term future sea-level rise do not implicate a global rise of 5 m, however the application of such a scenario is useful in highlighting the global extent and locations of low elevation peatlands. Indeed, the 1 km resolution of the DEM we applied allows the assumption that grid squares of 5 m average elevation include substantial land areas vulnerable to small increases in sea level. Also in the tropics where subsidence of already low lying coastal peatlands equivalent in magnitude to +5 m sea-level rise have been widely reported, the application of such a scenario is relevant for projecting change by 2100.

Unfortunately, a dataset of true peatland cover and distribution remains unavailable for many regions of the world^2^. Following this, we recognise that both estimations are made with a high degree of uncertainty stemming from a variety of sources (Table 3). Future refinements would benefit substantially from: accounting for global peat accretion and subsidence, modelling spatial variability in sea-level trends, mapping peatland extent with higher accuracy and by using more accurate and locally specific elevation datasets.

Once we take uncertainties into consideration, the impact of future global sea-level rise alone, at policy-relevant timescales, will be comparatively minor against other threats peatlands face. However, anthropogenic perturbation of natural peatlands (notably subsidence), will render some regions substantially more vulnerable to the threat of coastal inundation, even where absolute sea level does not change. Similarly other anthropogenic stresses on peatlands (e.g. shrinking of bioclimatic envelopes^16^) may increasingly mean that coastal peatlands either cannot keep pace with sea-level rise leading to eventual inundation, or cannot colonise areas exposed by falling sea levels, if these areas are no longer climatically suitable.

## Conclusion

Our results linking variable salt-input with changes in peatland ecosystem functioning across the unbroken succession of salt marsh to ombrotrophic blanket bog at Kentra Moss indicate that concentrated salt input has a significant negative effect on the rate of recent carbon accumulation. Therefore, inundation by sea-level rise in the future represents a threat to blanket bog and peatlands more generally.

Through altering both the vegetation assemblages which colonise the bog and also the decomposition processes taking place within the peat matrix, inputs of oceanic base cations alter the carbon balance, and therefore exert control on surface accretion and carbon accumulation rates. The level of inundation (tidally and by storm surges) constrains the coastward colonisation of *Sphagnum* mosses, key builders of high-latitude peatlands. Blanket bogs are however sufficiently flexible to persist in areas of moderate concentrations of salt-input, and this allows them to expand coastward where sea level and climatic conditions allow. We estimate that the current extent of blanket bogs at very low elevation (≤5 m above sea level) is 11,500 km^2^ equating to a carbon store of ca. 2.1 ± 0.34 Gt.

Under scenarios of sea-level rise, where blanket bogs are inundated by salt-water, we forecast that an inland shift of all sections (A, B and C) will occur, i.e. saltmarsh (halophilic) plant communities will colonise the peat substrate and decomposition processes will change. This will correspond to a decline in carbon accumulation compared to blanket bogs. Globally, the blanket bog area vulnerable to future salt-water inundation is equivalent to a total sink of 0.22 Tg C yr^−1^.

We estimate that ~145,000 km^2^ of global peatlands containing 20.2 ± 2.5 Gt C are located ≤5 m above sea level. Our mapping of these vulnerable areas, the first of its type, may therefore be used to aid higher accuracy and targeted assessments of peatland vulnerability at the regional scale. We provisionally show that tropical peatlands are proportionally at greater risk (~16.6% of total area, 61,000 km^2^ and 8.3 ± 1 Gt of stored carbon). Following this, the region where peatlands face the greatest risk of inundation is South-east Asia, especially Kalimantan and Sumatra, where recent land-use change is resulting in rapid peatland subsidence. Generally, tropical peatland areas will suffer disproportionately relative to those at high latitude. This is because high-latitude (>40°N) peatlands have colonised land that is more likely to be recently deglaciated and many of these deglaciated areas are experiencing isostatic rebound. Rapid and significant peat subsidence will also amplify the risk of salt-water inundation facing peatlands in the tropics.

The inundation of peat by salt-water does not necessarily mean that all stored carbon would be oxidised however the overall contribution of freshwater peatlands as sinks in the global carbon cycle would inevitably be reduced. The impacts of peatland inundation are likely to be regionally important, especially in the tropics. In such regions the value of peatlands extends beyond their function as carbon sinks but rather as providers for livelihoods, provision of land as well as other essential ecosystem services^41^,^45^. Further consideration of the controlling influence of the ocean on sustainable peatland carbon accumulation should focus on the longer-term impacts associated with anthropogenic perturbation of the salt-water inundation risk at time-scales analogous to peatland accretion.

## Methods

### Field Methodology

We established a 450 m wide transect belt across the blanket bog at Kentra Bay, which stretched 2 km inland from the coastal interface, and avoided areas of historical disturbance (Fig. 1). We avoided sampling in immediate proximity to the various rock intrusions (Supplementary material 1) or the road intersecting the site.

We recorded variability in surface water conductivity (salinity) with distance inland along two transects, using a calibrated Hanna probe (HI 98129) which compensated values to a standard temperature. Conductivity is linearly related to salinity under the measuring conditions at the site and therefore makes a good proxy for salinity^46^. Two-hundred measurements of conductivity were taken alongside measurements of pH, total dissolved solids and temperature. Sampling of every existing pool enhanced data coverage at the coastal interface (0–200 m inland) where most variability was identified.

Thirteen cores spaced along the transect were sampled using monolith tins to a depth of 30 cm. Cores were taken mid-range between mounds and hollows on the peatland surface to control micro-topographic heterogeneity in accumulation^47^. Compaction and sampling losses were controlled for both techniques by cutting surface vegetation with a serrated knife before extraction of the cores^48^. Cores were wrapped in plastic film and refrigerated prior to subsampling.

Peat depth measurements were taken by inserting a metal probe vertically from the surface until the point of obstruction, interpreted as the interface with the underlying fluvioglacial fan (sand and gravel). Three separate transects were used to account for local scale depth variability.

We recorded the percentage cover of vascular plants, *Sphagnum* and other bryophyte species within metre-square quadrats spaced along the transect belt. Vegetation (canopy) height was recorded by measuring individual species present before calculating a quadrat average based on the percentage cover of each species.

### Laboratory Methodology

Each monolith was subsampled at contiguous 1 cm resolution, ensuring constant sample volume (6.25 cm^3^). Bulk density was calculated by dividing sample mass after freeze-drying by volume^49^. Change of sample mass after drying determined the moisture content. Organic matter content was calculated from measurements of loss on ignition (550 °C for 4 hours) at 2 cm intervals, and converted to carbon content using a regression for British peat and organic soils^50^. Carbon density (gCcm^−3^) was calculated by multiplying bulk density with percentage carbon content^4^. Ash-free bulk density, a simple proxy for degree of peat decomposition, was derived from multiplying bulk density by organic matter content^25^,^49^.

A high-resolution chronology for five of the monolith cores was established using ^210^Pb dating. Samples were acid digested with concentrated HCl, HNO_3_, and H_2_O_2_, and a spike of ^209^Po added as a yield tracer. Material was plated onto silver disks and the activity measured by alpha spectrometry using an Ortec Octête Plus Integrated Alpha-Spectrometry System at the University of Exeter (UK) Radiometry Lab. Resulting activities were reported with 1-sigma analytical uncertainty. The ratio ^210^Po/^209^Po is calculated to derive the ^210^Pb activity, which is then corrected for sample size, decay rates and time between preparation and measurement. The date of each sample was derived from the ^210^Pb inventory using the constant rate of supply model^51^.

Due to leaching of ^210^Pb in sediments prone to significant water-table fluctuation^52^ Spheroidal Carbonaceous Particles (SCPs), a frequently applied technique^53^,^54^, were used as alternative dating features for the salt-marsh sediments proximal to the coast. SCP samples were prepared with a sequence of selective acid digestions^55^,^56^, and their presence within each sample counted under a microscope. SCP identification followed Rose (2008)^57^. Relative SCP concentrations per sample were deduced to identify two dating features; peak (1978 ± 4 AD) and initial take-off (1850 ± 25 AD)^54^. BACON, a flexible Bayesian age–depth modelling approach that uses prior information on likely accumulation rates and their plausible variability over time was used to produce an age-depth model from the results^58^. For all cores, three priors (the accumulation rate, section thickness and memory) were set to default values, (acc.shape = 1.5, acc.mean = 20, res = 5)^58^. Total carbon accumulation was calculated by identifying the depth of the year 1900, and totalling quantity of carbon within the core above (Supplementary material 6).

### Computational Methodology

#### Field Site

Individual datasets from Kentra Bay were synthesised using ArcMap 10.2. We used a 5 m grid resolution digital terrain model (Ordnance Survey Terrain 5) to measure elevation above sea level and calculate distance inland for each individual data point. We defined the coast as the boundary between marine sediment and the first salt-marsh plants.

#### Mapping low elevation peatlands

Global Peatland Cover Map: A map of freshwater peatland areas was obtained from Yu *et al*.^2^. The map comprises data obtained from individual regional inventories of soil type (see reference for individual data sources).

Global Blanket Bog Map: We created a map of global blanket bog area by integrating mapped areas of bioclimatic suitability^16^ with the Global Peatland Cover Map. Data for both peatland and blanket bog cover was converted to a 1 km cell grid to standardise resolution for analysis.

*Hydro1k Digital Elevation Model* (*DEM*) is a hydrologically adjusted derivative of the GTOPO30 elevation dataset, with global coverage (excluding Greenland and Antarctica) at 1 km resolution (available from the U.S. Geological Survey). We applied this dataset because; (1) it has harmonized sea levels, (2) it has fixed values for open water, and (3) it makes our estimate of vulnerable peatland area comparable with a previous estimate for organic soils^10^.

Using ArcMap 10.2, peatlands at 2, 5 and 10 m above sea level were isolated using elevation values derived from the DEM. This provided a numerical vulnerability score for each cell containing peatland and a corresponding total area of at risk peatlands. The same technique was applied for blanket bog areas. A correction factor was employed to equalise the global peatland area indicated by the peat map with accepted values from the literature^2^,^4^. Disparity in peatland coverage estimation arises because; (1) the map indicates peatland abundant regions (peat cover ≥5%) rather than true coverage^2^, (2) there is spatial variability within the inventories used to assemble the dataset, and (3) the differential classification of peatlands between countries.

## Additional Information

**How to cite this article**: Whittle, A. and Gallego-Sala, A. V. Vulnerability of the peatland carbon sink to sea level rise. *Sci. Rep*. **6**, 28758; doi: 10.1038/srep28758 (2016).

## Supplementary Material

Supplementary Information

## Figures and Tables

**Table 1:** Average (surface 30 cm) bulk density and percentage carbon for cores located along the Kentra Bay salinity gradient.

		Bulk Density (g cm^−3)^		Carbon Content (%)		Total Carbon Accumulated since 1900 (gCm^2^) /Rate (gCm^2^year^−1^)
Salinity gradient position and section		Mean	Standard Deviation	Mean	Standard Deviation	
Inundated by salt water (tidal)	A	0.3821 ± 0.0225 (30)	0.1234	18.83 ± 2.09 (15)	8.11	**4,849**/−42.17 (2)
Inundated infrequently (storms)	A	0.2934 ± 0.0225 (50)	0.1592	31.02 ± 2.79 (26)	14.25	
Not Inundated-Proximal to Coast	B	0.1274 ± 0.0028 (208)	0.0400	50.46 ± 0.14(123)	1.51	**7,384**/−64.21 (3)
Not Inundated-Distal from Coast	C	0.0705 ± 0.0031 (89)	0.0292	50.86 ± 0.10 (52)	0.71	**5,390**/−46.87 (3)

Values represent the mean ± SE with number of samples (bracketed). Negative values for rate of accumulation indicate net carbon sequestration from the atmosphere.

**Table 2:** Areas of low elevation blanket bogs and peatlands globally, with the percentage of current total extent potentially vulnerable to inundation.

Elevation (m)	Blanket Bog		All Peatlands		Tropical		Southern		Northern	
	Area (km^2^)	% Area	Area (km^2^)	% Area	Area (km^2^)	% Area	Area (km^2^)	% Area	Area (km^2^)	% Area
+2	8,406	5.42	98,313	2.23	47,888	13.00	2,235	4.97	48,190	1.20
+5	11,482	7.41	143,866	3.26	61,000	16.55	2,762	6.14	80,103	2.00
+10	15,795	10.19	304,715	6.90	123,238	33.44	6,279	13.99	190,829	4.77

**Table 3:** Sources of uncertainty associated with estimating the global extent of peatlands vulnerable to sea-level change.

Source of uncertainty	Description
*Mapping accuracy and resolution*	Mapping error and resolution associated with peatland extent, as well as land-surface heights may result in under or over estimation of areas at risk.
*Subsidence or Accretion of Peat*	Active peat accumulation (increasing land surface height) could outpace and limit the effect of sea-level rise, whilst subsidence (lowering) would amplify relative sea-level rise. A positive feedback could develop if inundation of peat caused further subsidence.
	Anthropogenic perturbation of healthy blanket bog functioning could complicate this balance.
*Type of coastline*	Peat soil has low mechanical strength to resist erosion, so loss of particulate organic carbon via direct erosion would amplify losses due to sea-level change on high-energy coasts.
	Enhanced salt-spray input may limit accumulation where direct contact does not occur. On low energy coastlines peat accumulation may persist.
	Failure of coastal management structures may result in unexpected flooding of peatland areas.
*Initiation of new peatland*	Sea-level rise could increase inland water tables resulting in peatland expansion, offsetting losses by inundation elsewhere. Exposure of land on emergent coasts may allow new peatland colonisation where suitable conditions exist.
*Use of global sea-level rise predictions*	High latitude landmasses have been recently deglaciated resulting in a complex pattern of global isostatic land-surface rebound. This can slow or reverse relative sea-level rise. Low-elevation peatlands in rebounding areas will be less vulnerable to inundation.
*Changes in storminess*	Increased storminess may result in dramatic ‘one-off’ rapid erosion events of coastal peatlands.
	Increased salt inputs, from spray and storm surges, may input sufficient oceanic salt to peatlands changing their ecosystem functioning and carbon sink potential^59^.
*Indirect salt water intrusion inland*	Salinisation of freshwater aquifers may cause ecosystem changes to peatlands even where direct inundation does not occur.
*Rapid land surface movements*	Extreme (unpredictable) events leading to rapid land surface movements and subsequent salt-water inundation of peatlands, such as volcanic eruptions, landslides and earthquakes^60^.
