# Supplementary material for: Vulnerability of the peatland carbon sink to sea-level rise
Source: Sci Rep. 2016 Jun 29;6:28758. doi: 10.1038/srep28758 (PMC4926159; doi:10.1038/srep28758)
Supplement: Supplementary Information [file srep28758-s1.pdf]

Vulnerability of the peatland carbon sink to sea-level rise.

Supplementary Material

Alex Whittle[1], and Angela V. Gallego-Sala[1,*]

[1]Department of Geography, University of Exeter, Exeter, EX4 4RJ, UK

[*]A.Gallego-Sala@exeter.ac.uk

Supplementary Material 1 – Study area context

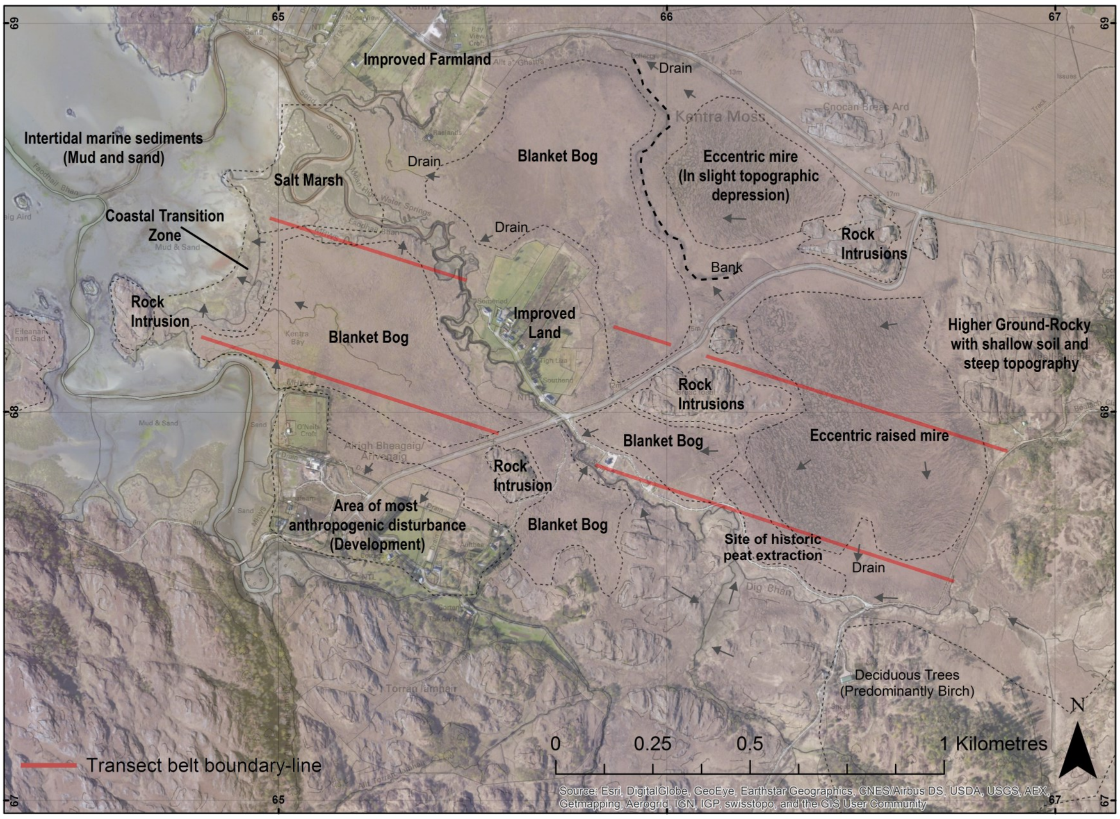

Major mesotopes comprising blanket bog surrounding our transect belt. Arrows indicate the perceived direction of water flow based on surface height and river flow made from field observations. Aerial imagery illustrates the pronounced surface patterning of pools and elongate ridges, a product of underlying topography. Produced using ESRI ArcMap 10.1. (http://www.esri.com/). Contains OS data © Crown copyright (2015).

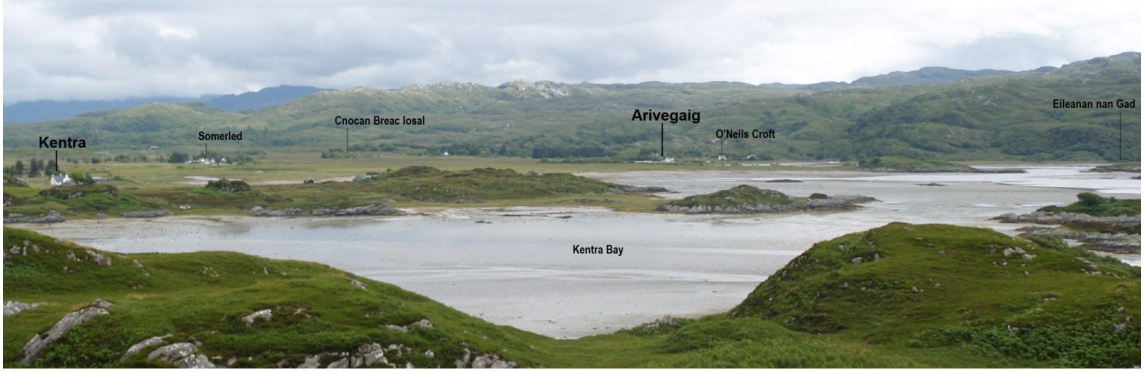

Photograph looking Southwards across Kentra Bay and Moss demonstrating the slight gradient East-to-west toward the Bay. Also shows the rocky outcrops which protrude from the blanket bog and the overall low elevation of the area, which make the bog vulnerable to storm surge inundation. Source: Alex Whittle.

Supplementary Material 2 – Variability in surface water chemistry

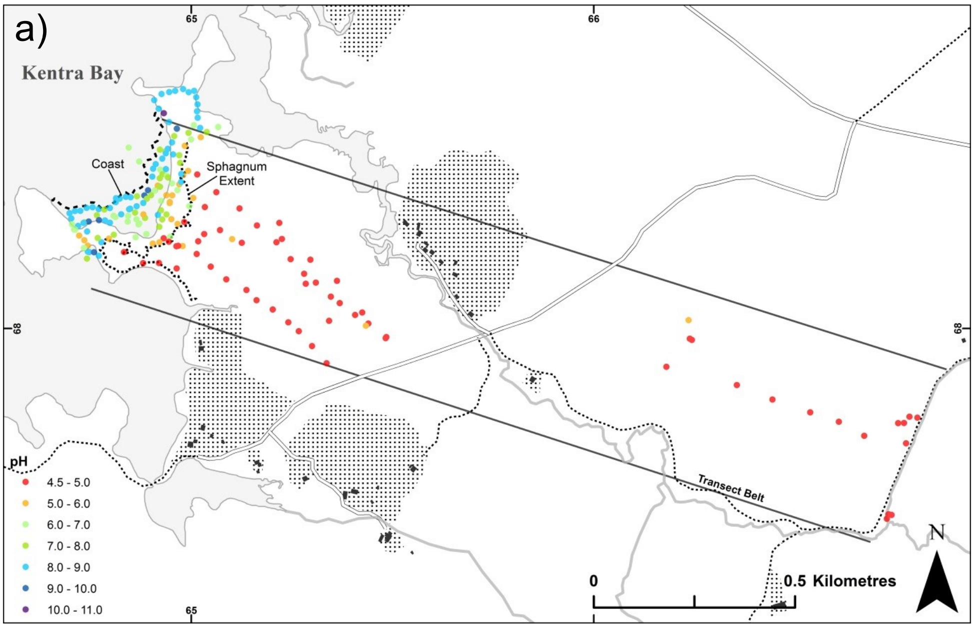

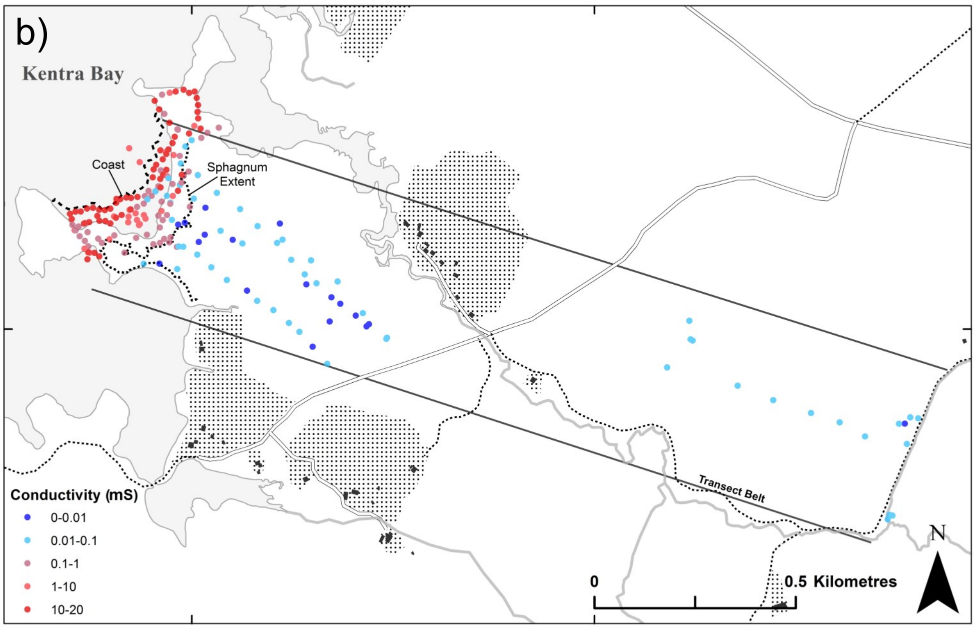

Surface water pH (a) and Conductivity (b) within the transect belt used in this report. Position of the coast is defined as the boundary between marine sediments and first terrestrial plants. Conductivity values are shown on a log scale where 20 mS was the maximum recordable value with the meter used. Produced using ESRI ArcMap 10.1. (http://www.esri.com/). Contains OS data © Crown copyright (2015).

Supplementary Material 3 – Transect Photographs

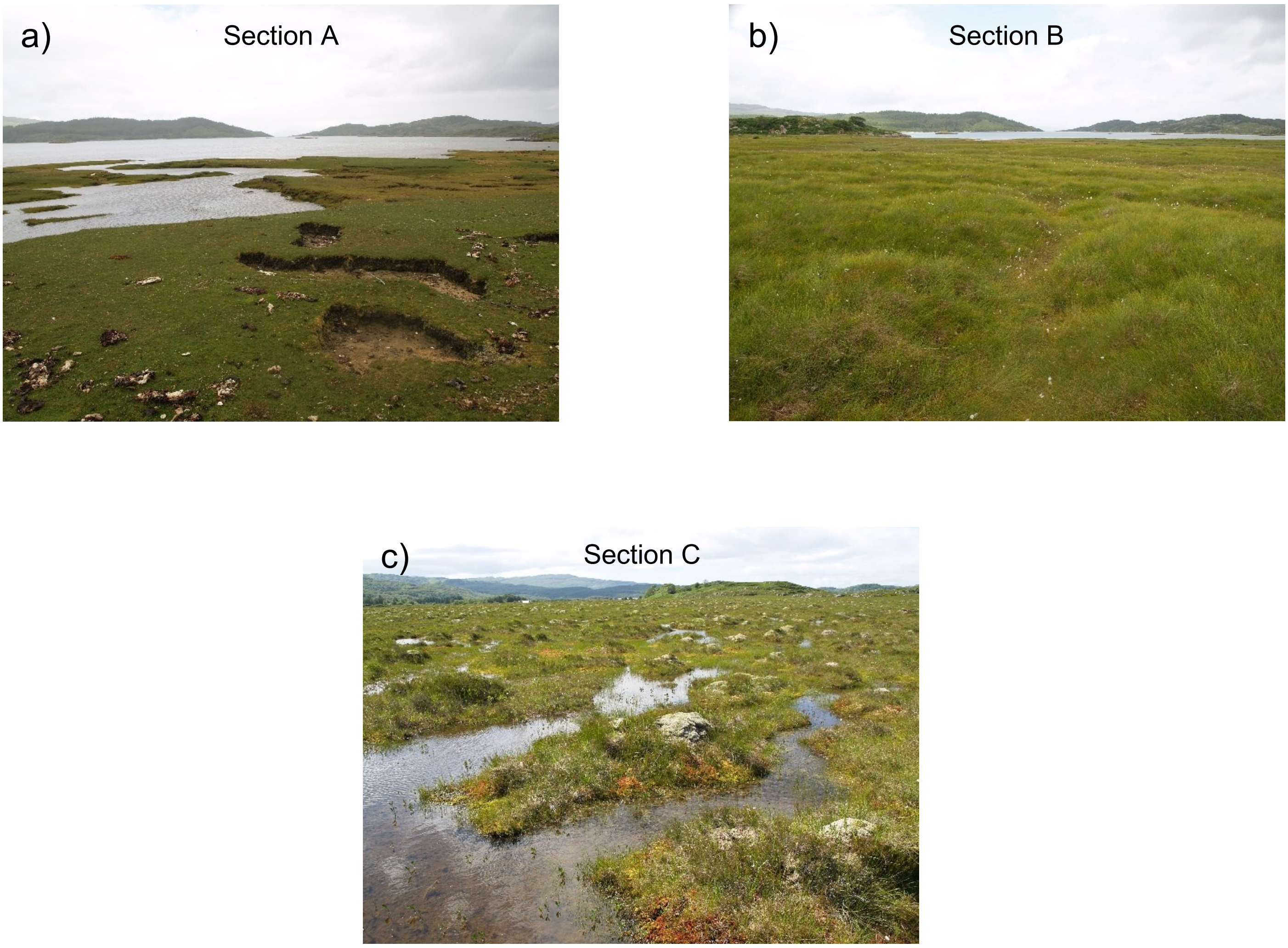

Photographs of the bog surface within each section of salt influence; (a) inundated land at the coastal interface, (b) areas close to the coast but not inundated, and (c) areas isolated from the coast approximately 2 km inland. Note that all photographs are taken in the same direction toward the coast. Source: Alex Whittle.

Supplementary Material 4 – Kentra Bay inundation modelling

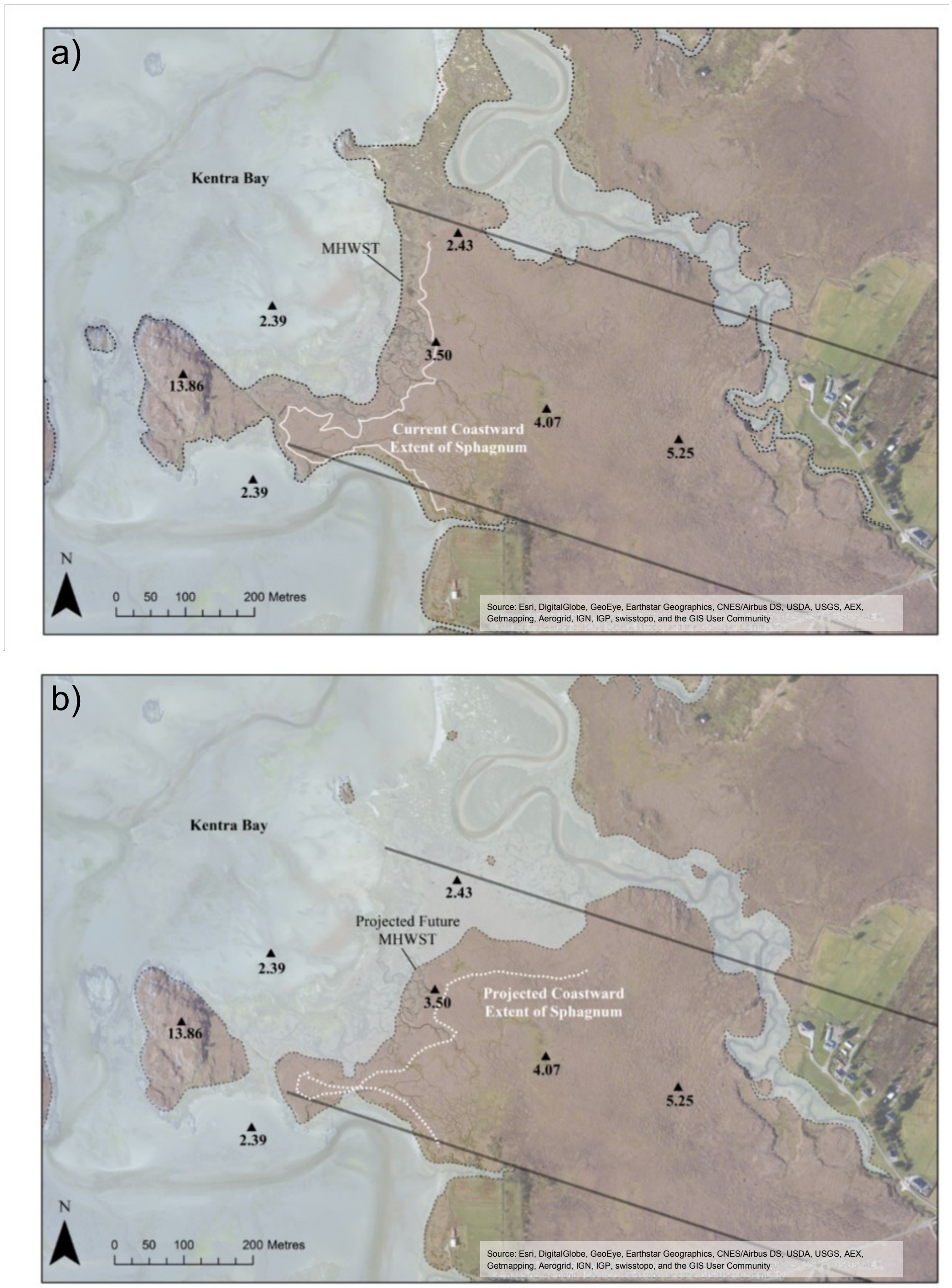

Projected impact of future sea-level rise on the coastal interface at Kentra Moss (a) showing the current level of mean high water spring tide (MHWST) and observed maximum coastward extent of *Sphagnum* clumps (>30 cm diameter). (b) Level of inundation based on +30 cm sea-level rise by 2100. Modelled water level assumes a constant rate of accretion within the coastal zone (~+0.64 cm/decade) but does not account for erosion or compaction of the peat surface which may occur under increased inundation frequencies. Spot heights (m above ordinance datum) are given for reference, as well as a projection for future *Sphagnum* extent derived from its current average position relative to the coast. Produced using ESRI ArcMap 10.1. (http://www.esri.com/). Contains OS data © Crown copyright (2015).

Supplementary Material 5 – Peatlands ≤5 m above sea level in South-east Asia.

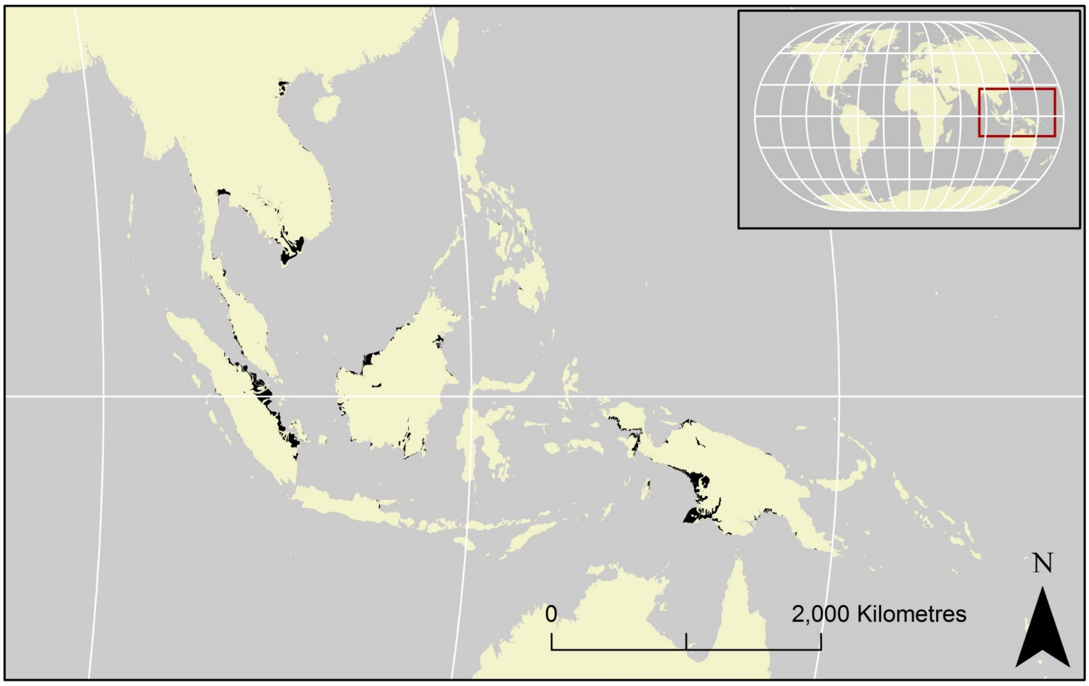

Low elevation peatlands in South-east Asia (black shading). Areas isolated from the coast indicated as lying ≤5 m, are likely to result in the overlap of peatland extent mapping with open waters which are set to 2 m in the Hydro1k DEM. Produced using ESRI ArcMap 10.2. (http://www.esri.com/).

Supplementary Material 6 – Age-depth models and carbon inventories for analysed cores.

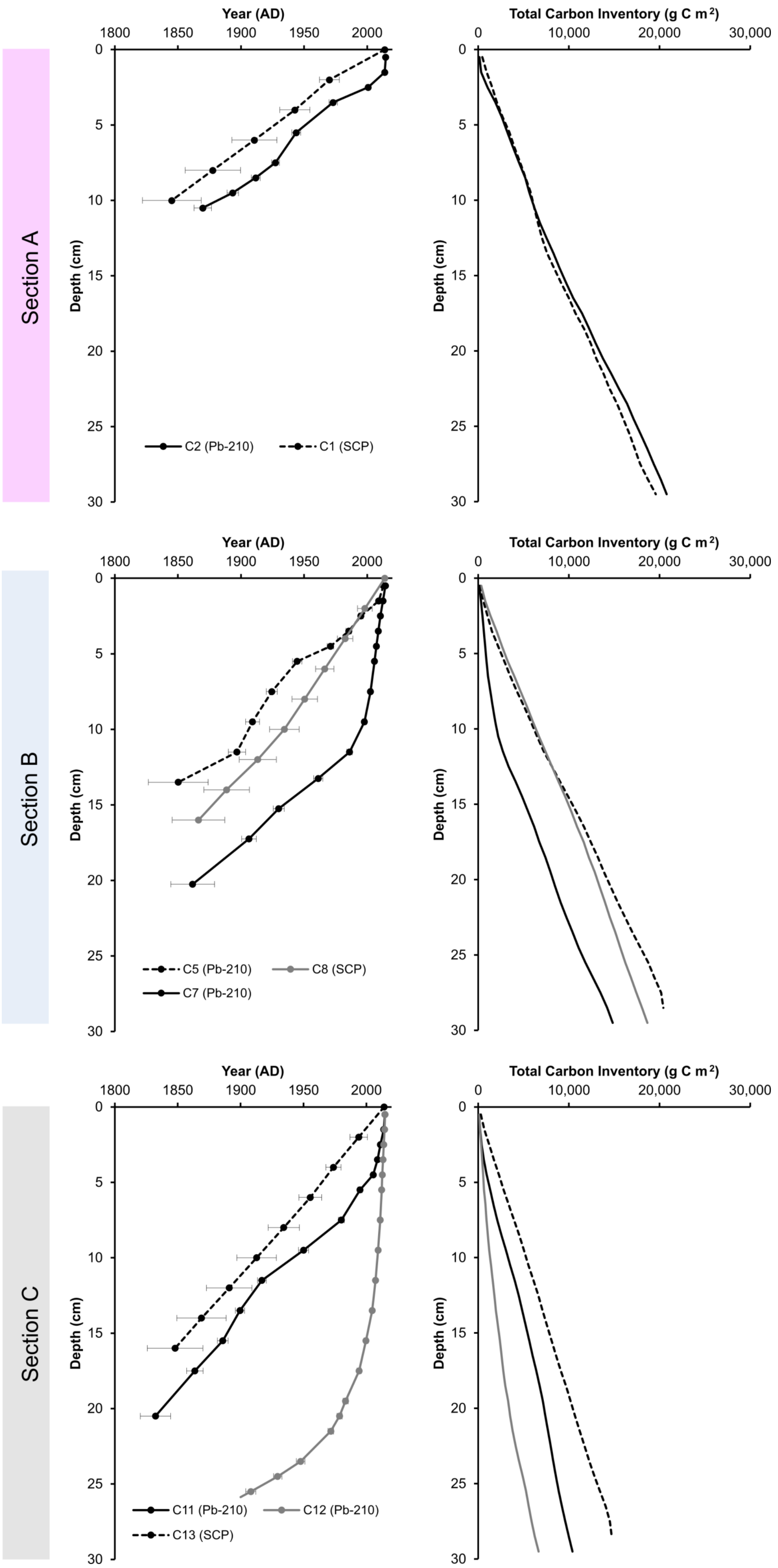

Age-depth models and corresponding carbon inventories for each section of the coastal marsh to blanket bog transect. Section A-C correspond with designations throughout the text. Error bars on age-depth models for both ^{210}Pb and SCP dating show 1-sigma dating uncertainty. The location of each core are shown in Fig. 1.
